# Small-Molecule Therapeutic Perspectives for the Treatment of Progeria

**DOI:** 10.3390/ijms22137190

**Published:** 2021-07-03

**Authors:** Jon Macicior, Beatriz Marcos-Ramiro, Silvia Ortega-Gutiérrez

**Affiliations:** Departamento de Química Orgánica, Facultad de Ciencias Químicas, Universidad Complutense de Madrid, E-28040 Madrid, Spain; jonmacic@ucm.es (J.M.); emarcos@ucm.es (B.M.-R.)

**Keywords:** rare diseases, Hutchinson–Gilford progeria syndrome (HGPS), progeria, progerin, lamin A

## Abstract

Hutchinson–Gilford progeria syndrome (HGPS), or progeria, is an extremely rare disorder that belongs to the class of laminopathies, diseases characterized by alterations in the genes that encode for the lamin proteins or for their associated interacting proteins. In particular, progeria is caused by a point mutation in the gene that codifies for the lamin A gene. This mutation ultimately leads to the biosynthesis of a mutated version of lamin A called progerin, which accumulates abnormally in the nuclear lamina. This accumulation elicits several alterations at the nuclear, cellular, and tissue levels that are phenotypically reflected in a systemic disorder with important alterations, mainly in the cardiovascular system, bones, skin, and overall growth, which results in premature death at an average age of 14.5 years. In 2020, lonafarnib became the first (and only) FDA approved drug for treating progeria. In this context, the present review focuses on the different therapeutic strategies currently under development, with special attention to the new small molecules described in recent years, which may represent the upcoming first-in-class drugs with new mechanisms of action endowed with effectiveness not only to treat but also to cure progeria.

## 1. Introduction

Hutchinson–Gilford progeria syndrome (HGPS), or progeria, is one of the rarest diseases within the rare disease field. It affects around 1 in 4 million new births, and no predisposition factor has been found, so it is considered as sporadic. Unlike the majority of the rare diseases, it has, since November 2020, a specific FDA approved drug, lonafarnib. However, this small molecule represents a treatment, but it does not cure the disease, and it has several limitations that make the development of new therapeutic strategies a critical need in the field. In this context, in the present review we analyze the different and more recent pharmacological approaches that probably will lead to new therapies in the upcoming years. Starting by summarizing the molecular causes of the disease and their implications at the organism level, together with the available animal models, we examine the different options for pharmacological intervention (at the DNA or gene level, at the RNA or messenger level, and finally at the protein level) with a special focus on the small molecule field and the different mechanisms of action that could yield new first-in-class drugs in the next few years. Then, we provide up to date information of the ongoing clinical trials and our vision for the future directions of the field.

### 1.1. Hutchison–Gilford Progeria Syndrome and Its Molecular Causes

**Figure 1:**
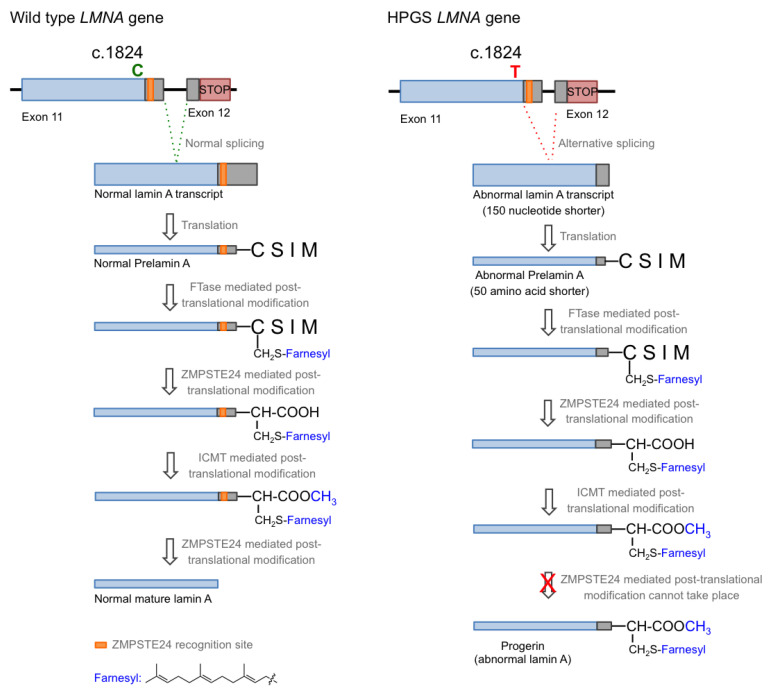
Biosynthesis of normal mature lamin A (**left**) and progerin (**right**).

Hutchison–Gilford progeria syndrome (HGPS), or progeria, is a rare human genetic disorder caused by a specific mutation in the *LMNA* gene, which codes for lamins A and C. This autosomal dominant disease is triggered by the single nucleotide substitution mutation c.1824C > T in exon 11. Although this mutation should not entail any consequence since both nucleotide triplets (wild type and mutant) code for glycine (p.G608G mutation), the mutation activates a cryptic donor splicing site for lamin A-specific transcript processing, leaving the splicing for the lamin C transcript unaffected (see Figure 1 for details) [1,2,3]. The appearance of this new splicing site in the lamin A gene leads to the generation of a messenger RNA with a missing part, resulting in the production of a mutated form of lamin A, which is called progerin. Progerin lacks 50 amino acid residues encoded by the lost fragment in exon 11. This deletion is critical because it contains a recognition site for ZMPSTE24 cysteine proteinase, which is involved in the last step of the post-translational processing of prelamin A protein. In normal cells, prelamin A, as expected from the CAAX sequence in its C-terminus, is subjected to a specific set of post-translational modifications. These transformations include the following stages: (i) farnesylation of the cysteine residue; (ii) cleavage of the AAX terminal tripeptide; (iii) carboxymethylation of the C-terminal cysteine; and (iv) final excision of the last 15 amino acids due to the existence of a specific recognition site. The first step is catalyzed by the enzyme farnesyl transferase (FTase), the two hydrolytic events are mediated by ZMPSTE24, and the methylation reaction is carried out by isoprenylcysteine carboxylmethyltransferase (ICMT). In progerin, once the methylation step has occurred, the last peptide removal cannot occur due to the lack of the ZMPSTE24 recognition site, so the produced protein is a 15 amino acid longer, permanently farnesylated and methylated version of lamin A (Figure 1) [4].

HGPS belongs to a broader group of diseases called laminopathies [5], which are a consequence of different mutations in the LMNA gene. All of them result in a wide spectrum of overlapping disorders that comprise muscular dystrophies, a peripheral neuropathy, lipodystrophy syndromes, and accelerated aging disorders that share some of the features of progeria [6]. In addition, there are other progeria-related phenotypes referred to as non-classical mutations that are frequently described as progeroid laminopathies or atypical progeroid syndromes. These disorders have been described in detail elsewhere [7,8,9], so in this review, we will focus on classical HGPS (p.G608G mutation), which has only an autosomal dominant mode of inheritance and a clearly defined molecular background.

### 1.2. Progeria Phenotype: Nuclear, Cell, and Tissue Defects

**Figure 2:**
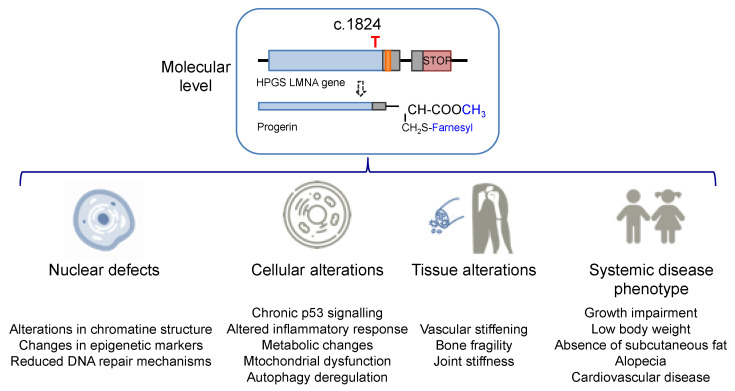
Alterations in HGPS individuals from the molecular and cellular levels to the disease phenotype.

The increase in hydrophobicity triggers the abnormal accumulation of progerin in the nuclear membrane. This leads to the appearance of many cell defects, especially nuclear alterations, as would be expected from the fundamental structural role that lamin A plays in the nucleus. Progeroid cells display anomalous nuclear morphology and impaired nuclear functions, fundamentally due to the lack of the dynamic movement that characterizes the healthy lamin, which shifts between the nuclear lamina polymer in the nuclear lamina and the nucleoplasm [10]. Progerin, however, is strongly attached to the lamin because of its high hydrophobicity, and it is not able of participate in this dynamic cycle. This fact provokes a thickening of the lamina, enlarges cellular stiffness, and impairs many nuclear functions [11,12]. Increased cellular rigidity seems to be central in HGPS, as the disease affects mainly cells that must respond to changes in mechanical stress such as vascular cells, bone, and joints, and these three tissues recapitulate some of the most prominent disease symptoms in progeria patients [13,14]. Other remarkable nuclear defects in HGPS cells are related to DNA and chromosome functions. They include extensive alterations in chromatin structure, as reflected in the loss of heterochromatin domains, changes in epigenetic markers, and reduced DNA repair mechanisms [15,16]. These deficiencies translate into global cell defects that include chronic p53 signaling, altered inflammatory response, metabolic changes, autophagy deregulation, and stem cell dysfunction [2,17]. Altogether, they are eventually responsible for the complex and distinctive disease phenotype, characterized by growth impairment, low body weight, absence of subcutaneous fat, lipodystrophy, decreased joint mobility, alopecia, and cardiovascular disease, features that reflect in the overall premature aged aspect of diseased children (see Figure 2 for an schematic summary). Average life expectancy is about 14.6 years, and the main direct cause of death is cardiovascular disease (CVD) characterized by atherosclerosis, vascular stiffening and calcification, electrocardiographic alterations, and left ventricular diastolic dysfunction, and derived complications [14,18]. 

### 1.3. Animal Models

As in any other disease, the availability of cell and animal disease models is key to the translation of any potential preclinical therapy into humans. Despite their limitations, HGPS mouse models have been critical to decide which therapies should be tested in patients. Available HGPS mouse models either ectopically express progerin, lack or over-express A-type lamin isoforms, or accumulate farnesylated prelamin A. Up to this moment, different *LMNA* and *ZMPSTE24* genetically modified models have been described. One of the first mouse models to document a prelamin A processing defect was the *Zmpste24^−/−^* mouse, characterized by the development of kyphosis and spontaneous bone fractures in multiple locations [19,20]. However, these mice only showed partial heart alterations that are characteristic of progeria patients [21], a fact that highlights the need for additional mouse models that mirror the disease phenotype more completely. In this regard, during the following years, different models were developed, such as a mouse heterozygous for a human transgene with the G608G mutation in *LMNA* (p.G608G/+). This mouse exhibited the progressive vascular abnormalities that are the main causes of lethality in HGPS patients [22] but lacked the rest of the phenotypic features observed in human patients. In order to fill this gap and better recapitulate all the alterations present in HGPS patients, a *Lmna^G609G/G609G^* homozygous animal model was generated. These mice accumulate progerin, present histological and transcriptional alterations characteristic of progeroid models, and phenocopy the main clinical manifestations of human HGPS, including shortened life span and bone and cardiovascular aberrations, and probably represent the most reliable progeria mouse model [23]. More recently, the G608G transgene model was bred to homozygosity, resulting in an HGPS mouse model that replicates many aspects of both musculoskeletal and vascular changes of HGPS human patients [24].

Although these mice recapitulate many aspects of the disease, none of them has been reported to develop atherosclerosis, the most single life-threatening factor of HGPS. Within this aim, the *Apoe^−/−^Lmna^G609G/G609G^* mouse model was generated [25]. These mice ubiquitously express progerin and display the premature aging phenotype together with an accelerated atherosclerosis. Furthermore, to precisely delineate the contribution of different cell types to the development of atherosclerosis, *Apoe^−/−^Lmna^LCS/LCS^* mice were crossed with LysMCre and SM22αCre mice, respectively, to induce progerin expression specifically in macrophages or vascular smooth muscle cells (VSMCs). This study suggests that progerin-expressing VSMCs play an important role in triggering atherosclerosis, plaque vulnerability, and eventual death [25]. Additionally, an endothelium-specific HGPS mouse model with selective endothelial progerin expression points out the contribution of progerin-expressing endothelial cells to the fibrosis and cardiovascular disease observed in HGPS patients [26]. 

These mouse models have undoubtedly contributed to the clinical translation of the only drug currently approved for progeria treatment. However, the availability of additional animal models, more similar to humans, could help in the prioritization of those experimental therapies with higher success probability. Towards this aim, the first large animal model for HPGS has been recently generated using CRISPR-Cas9 gene editing. This knockin heterozygous *LMNA c.1824C > T* Yucatan minipig displays the main cellular and phenotypic features observed in human patients, such as the expression of progerin and normal lamin A/C, growth retardation, lipodystrophy, skin and bone alterations, cardiovascular alterations, cardiovascular disease, and mortality around puberty [27]. This model can facilitate the translation of mouse preclinical research into human therapies by providing a more adequate model to investigate human-size interventional devices and to optimize pharmacological therapies before advancing to clinical trials, thus accelerating the development of effective therapies for HGPS patients. Table 1 summarizes the main features and limitations of the currently available progeria animal models.

## 2. Therapeutic Strategies for Treating Progeria

The identification of the molecular cause of the disease two decades ago spurred the quest for an effective pharmacological treatment for progeria. Within this overall and ambitious aim, different strategies have received attention [28,29]. They can be broadly classified into three main groups: (a) gene therapy approaches; (b) development of biologicals; and (c) small molecule treatments. Although this review is focused mainly on the third category, the two first will be briefly described to contextualize the field. 

### 2.1. Gene Therapy Approaches 

Gene therapy approaches have the indisputable advantage of selectivity and specificity, as they are aimed at the correction of the root of the problem, that is, to directly repair the disease-causing mutation. This therapy still has many practical limitations that preclude its use in the short term, but recent developments suggest it as a valuable possibility in the future. The current status of this technology and its application to HGPS has been reviewed recently [30], but some recent advances deserve special attention. In 2019, two independent studies demonstrated that interfering with lamin A/progerin expression by targeting *LMNA* exon with the CRISPR/Cas9-based genome-editing approach significantly improves the overall progeroid phenotype amelioration and extends lifespan [31,32]. However, this strategy affects lamin A expression together with progerin expression, and although lamin A is dispensable in cells and mice, the consequences of abrogating its expression in humans remain unexplored. In addition, the resulting diversity of uncharacterized insertion and deletion products at the target locus together with the risk of disrupting the wild-type *LMNA* allele, which differs only at a single base pair from the pathogenic allele pose challenges to clinical translation of this type of approach. Alternatively, a very recent study has used base editors, genome editing agents that directly convert targeted base pairs without making double-strand DNA breaks. Specifically, the use of adenine base editors (ABEs), which convert A•T to G•C, has allowed the correction of the *LMNA c.1824 C > T* mutation in fibroblasts derived from children with HGPS and in a mouse model. Lentiviral delivery of the ABE to human progeroid fibroblasts resulted in around 90% correction of the pathogenic allele, reduction of RNA mis-splicing, and progerin levels and correction of nuclear abnormalities. In the HGPS mouse model, a single retro-orbital injection of adeno-associated virus 9 (AAV9) encoding the ABE resulted in a remarkable correction of the pathogenic mutation, including restitution of normal RNA splicing and reduction of progerin levels. ABE administration increased the number of VSMCs and prevented fibrosis, two important markers of cardiovascular damage in progeria. Notably, a single injection of ABE-expressing AAV9 at postnatal day 14 improved vitality and greatly extended the median lifespan of the mice from 215 to 510 days, the highest increase observed with any experimental therapy to date [33]. In spite of this remarkable and encouraging result, which clearly suggests the potential of ABE base editors as a treatment for progeria, some limitations, such as the possibility of exacerbated immune responses or the induction of liver tumors, need to be addressed before this therapy can be applied to patients. 

### 2.2. Biologicals 

Biological drugs include a diverse group of products and are generally large, complex molecules such as nucleic acids, peptides, proteins, monoclonal antibodies, or vaccines. In the field of progeria, the antisense oligonucleotides (ASOs) are the most advanced candidates within this category. Structurally, ASOs are short oligonucleotides (10–20 monomers) that have a complementary sequence to the target RNA. Hence, the ASO will bind specifically to the target RNA by Watson–Crick base pairing rules and will avoid its translation by the ribosome, reducing the expression of the target protein. However, the use of unmodified ASOs, bearing a phosphodiester backbone, has important drawbacks, such as the lack of biological stability due to nuclease degradation or the limited passive diffusion because of the large size and negative charge. Hence, different chemical modifications aimed at increasing efficacy and stability and decreasing immune response and off-target toxicity have been described [34,35]. One of these modifications is the use of morpholino oligonucleotides, which are polymers constituted by the DNA standard nucleobases attached to a backbone of methylenemorpholine rings linked through phosphorodiamidate groups. In 2005, the first morpholino oligonucleotide complementary to the region containing the HGPS mutation in exon 11 was reported. This 25-mer oligonucleotide was able to correct aberrant splicing in different cell types, including dermal fibroblast and B-lymphocyte cell lines derived from HGPS patients, leading to a significant decrease in the levels of progerin in these cells. In spite of these promising results, the in vivo efficacy of this strategy could not be tested due to the lack of a suitable animal model, which was unavailable at that time [36]. Some years later, in 2011, the development of the specific *Lmna^G609G/G609G^* progeroid mice by the group of López-Otín made it possible to advance this concept with the report of specific vivo-morpholinos (morpholinos covalently attached to an octaguanidine dendrimer to facilitate efficient delivery into cells) capable of reducing progerin levels in vivo. This reduction paralleled an improved overall phenotype and corrected the main molecular and in vivo alterations characteristic of progeria. In particular, vivo-morpholino treatment significantly reduced the expression of p53 target genes as a marker of the senescent phenotype, normalized blood glucose levels, expanded the life expectancy of *Lmna^G609G/G609G^* mice, and improved body weight and lordokyphosis without noticeable toxic effects [23]. However, the cardiovascular pathology, critical in progeria, was not remarkably improved by administration of this morpholino oligonucleotide, a situation that has spurred research on this area, mainly aimed at optimizing bioavailability and in vivo efficacy of ASOs. Within this aim, the Misteli’s group addressed the de novo identification of ASOs using an unbiased approach based on the screening of a large diverse library of molecules with distinct target sequences, backbone chemistry, and lengths. This strategy has identified the ASO B143, which targets the *LMNA* exon 12 junction (instead of the mutated exonic splice site in exon 11, which was the target site of all the previously reported ASOs) and acts via non-RNase H-mediated mechanisms. To enhance cellular uptake and stability, B143 was conjugated with a palmitoyl acid chain. In vivo administration of this lipid modified ASO, named L-B143, induced a robust reduction in progerin mRNA levels in all tissues, but the extent of progerin protein reduction differed between organs, a result that suggests a long half-life and tissue-specific turnover of progerin in vivo [37]. Nonetheless, L-B143 produced a significant extension of lifespan in the transgenic mouse model of HGPS that expresses the human *LMNA* gene with the classic G608G mutation when administered subcutaneously at doses of 17 mg/kg or 50 mg/kg, although some toxicity concerns were reported at the highest dose. In this case, the improvement in survival and overall increase in body weight in treated animals run parallel to a reduction in incidence and severity of progeria-induced hypertrophy of the media in interstitial arteries of the heart, but it did not significantly correct aortic morphology [37]. At the same time, an independent study carried out by Collin’s group analyzed a series of phosphorodiamidate morpholino oligomers (PMOs) directed against the exon 11 cryptic splice site in five nucleotide intervals. Then, the best PMOs were conjugated to a peptide tag to enhance cell penetration and enable the in vivo intravenous administration of the compound for testing efficacy. This approach led to the identification of the peptide-conjugated PMO SRP-2001, which induced the highest decrease in progerin mRNA levels in patient fibroblasts. Intravenous delivery of SRP-2001 (60 mg/kg twice a week) to the transgenic mouse model of HGPS that expresses the human *LMNA* gene harboring the classic G608G mutation, induced around 60% increase in lifespan and reversed the loss of vascular smooth muscle cells in large arteries [38]. Together, these results highlight the potential of this therapeutic strategy for treating progeria.

### 2.3. Small Molecules 

In spite of the enormous advances in therapies based on gene editing strategies or ASOs, small molecules still endure as the most straightforward candidates for generating a specific therapy for progeria in the short term, as actually demonstrated by lonafarnib, the recently (and only) approved drug for this disease. Based on their different mechanisms of actions, small molecules can be classified into the following classes: (i) inhibitors of the prenylation pathway; (ii) methylation inhibitors; (iii) lamin A binders; and (iv) modulators of the downstream deleterious effects linked to progerin accumulation.

#### 2.3.1. Inhibitors of the Prenylation Pathway

**Figure 3:**
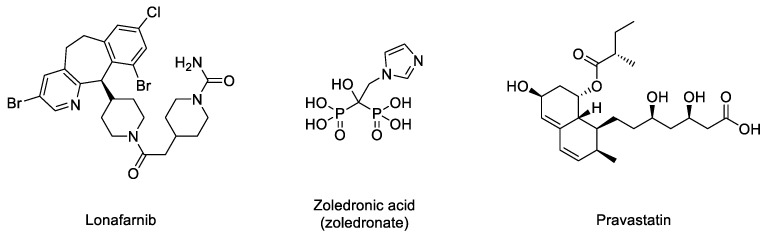
Structures of lonafarnib, zoledronic acid, and pravastatin.

In the normal post-translational prelamin A maturation process, the cytosolic enzyme FTase adds a 15-carbon farnesyl lipid to prelamin A that is eventually removed after the four-step translational processing suffered by this protein, schematized in Figure 1. However, the presence of the mutation prevents the last hydrolytic cleavage (Figure 1) yielding permanently farnesylated progerin. The presence of this group increases the hydrophobicity of the protein, thus promoting strong interactions with the nuclear membrane, where its abnormal accumulation severely affects the normal nuclear functions. Early studies described that blocking or preventing farnesylation improved nuclear abnormalities in mouse and human progeroid fibroblasts and reduced nuclear blebbing and the number of misshapen nuclei [11,39,40]. Furthermore, in vivo treatment with an FTase inhibitor (FTI) ameliorated the progeria phenotype in the Zmpste24-deficient mouse model of progeria [41]. Accordingly, inhibition of FTase was one of the first therapeutic strategies suggested for ameliorating the severity of the disease [42] and the only one that, up to date, has allowed the first approved drug for the specific treatment of progeria to be developed. FTIs were initially developed as anticancer compounds, aimed at preventing the permanent Ras activation characteristic of the frequent and deadly Ras-driven tumors [43]. The rational of this therapeutic potential was based on the fact that in the absence of farnesylation, Ras would be unable to attach to the cell membrane [44]. Hence, in the presence of an FTI, Ras should be inactive. Although the lack of efficacy of FTIs in phase III clinical trials stopped their advance into the clinic, the similarity between the post-translational processing of progerin and Ras (both belong to the CAAX family of proteins) set up the bases for the repurposing of FTIs for treating progeria by reducing the levels of farnesylated progerin. Among the different FTIs showing high efficacy in the inhibition of FTase in vivo, lonafarnib (Figure 3) soon stood out as the most promising candidate to start a single-arm phase II clinical trial in 2007 (NCT00425607) [45]. This trial showed that lonafarnib was well tolerated, and it also showed the capacity of this compound to improve some of the symptoms of the disease, such as rate of weight gain, arterial pulse wave velocity, carotid artery echodensity, skeletal rigidity, and sensorineural hearing. Importantly, it also decreased mortality rate (3.7% vs. 33.3% after a median of 2.2 years of follow-up in individuals receiving lonafarnib monotherapy compared with no treatment) [46]. Although lonafarnib does not correct all the alterations of the disease, such as lipodystrophy, skin features, alopecia, and joint contractures, the fact that it is the only available drug for treating this lethal pathology and that it has a positive impact in ameliorating specific features of HGPS, has led to its recent approval by the FDA under the name of Zokinvy^TM^ [47].

There may be various reasons for lonafarnib’s limited improvement. One of the first hypotheses suggested that in the absence of FTase activity, alternative prenylation by the enzyme geranyl geranyltransferase (GGTase) could take place, as this mechanism had been already characterized in the limited Ras inactivation observed in vivo after treatment with FTIs. Hence, it was suggested that the concomitant inhibition of both enzymes could increase the efficacy of the treatment. The phenotypic improvement observed in an in vivo model of progeria after the combination of the statin pravastatin and the aminobisphosphonate zoledronate seemed to confirm this idea [48] and was the basis for starting a triple therapy clinical trial in which lonafarnib, pravastatin, and zoledronic acid were administered to progeria patients (clinical trials NCT00879034 and NCT00916747). Although no participants withdrew because of side effects, no significant improvements other than increased bone mineral density were observed compared to lonafarnib monotherapy. Hence, as the triple therapy did not convey any obvious advantage over monotherapy, it was discontinued, and the clinical trial was prolonged but only with lonafarnib administration [49]. In addition, it is possible that other targets of FTase, apart from progerin, are affected by lonafarnib, eliciting adverse effects in progeroid patients. In line with this concern, since lonafarnib was originally developed for the treatment of Ras-dependent tumors [44], it is antiproliferative, a feature that can potentially limit its positive effects on progeroid cells, in which pro-proliferative effects are needed. In this regard, it has been reported that after long-term treatments, lonafarnib induced cell death and formation of donut-shaped nuclei [50,51]. These negative effects should be taken into consideration, especially when analyzing the effects of combining lonafarnib with other potential anti-progeroid therapies [51]. 

#### 2.3.2. Methylation Inhibitors

**Figure 4:**
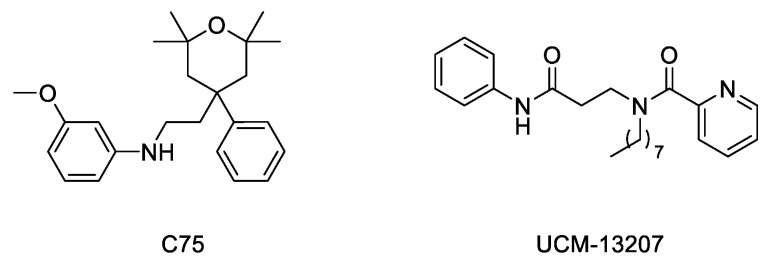
Structure of ICMT inhibitors C75 and UCM-13207.

Considering the importance of the farnesyl and methyl groups in increasing the lipophilicity of progerin and the efficacy of the FTI lonafarnib to improve the phenotype of the disease, the possibility that reducing methylation could reduce the abnormal accumulation of progerin in the nuclear membrane and hence improve the phenotype of the disease has received increasing attention. The importance of carboxyl methylation for proper membrane localization has been already described for other CAAX proteins, such as Ras [52,53]. In addition, inhibition of ICMT has been described as a promising strategy to inactivate Ras by inducing its mislocalization from the cellular membrane [54,55,56]. In this context, the finding that genetic disruption of ICMT improved the phenotype in the ZMPSTE24 mouse model of progeria supported that a small molecule ICMT inhibitor could represent a new therapeutic strategy to address HGPS. Within this aim, recent studies have provided strong evidence for the potential of ICMT inhibitors to improve the disease phenotype both in cellular and animal models. In particular, an optimized ICMT inhibitor, UCM-13207 (Figure 4), was able to increase cell viability, delocalize progerin from the nuclear membrane, and decrease DNA damage in cells from progeroid mice as well as in human fibroblasts from HGPS patients. In addition, this compound showed excellent efficacy in the in vivo progeroid mouse model *Lmna^G609G/G609G^*, where the compound increased body weight, enhanced grip strength, extended lifespan by 20%, and decreased tissue senescence in different organs together with key cardiovascular hallmarks [57,58]. In further support of the potential of this strategy, another ICMT inhibitor, compound C75 (Figure 4), was able to delay senescence and stimulate proliferation of human HGPS fibroblasts and to mislocalize progerin from the nuclear membrane towards the nucleoplasm, although no in vivo efficacy data were reported, probably due to limited pharmacokinetic properties of the compound [59]. Together, these results support the potential of the ICMT inhibitors, by themselves or in combination therapies, for treating progeria.

#### 2.3.3. Inhibitors of Progerin–Lamin A Interaction 

**Figure 5:**
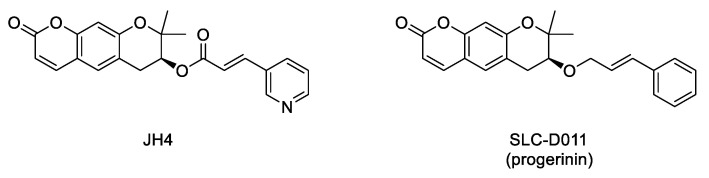
Structure of the inhibitors of the interaction between progerin and lamin A.

Interaction between progerin and lamin A has been established as critical for the development of the senescence phenotype associated with HGPS cells. Hence, it is conceivable that an inhibitor of this protein–protein interaction could improve the progeroid phenotype. Within this aim, an ELISA-based screening was carried out to identify progerin–lamin A interaction inhibitors. This first study succeeded in the characterization of JH4 (Figure 5) as a potent and specific inhibitor of this interaction. This compound improved nuclear deformation and senescence markers of progeroid cells. Remarkably, its administration (10 mg/Kg, intraperitoneally, twice a week) to *Lmna^G609G/G609G^* progeroid mice significantly ameliorated some of the characteristic features of progeria, including increase of body weight, grab strength, skin thickness, and improved nuclear deformation. In addition, administration of JH4 was able to extend lifespan in progeroid mice and to restore senescence-related markers [60]. However, the compound half-life after oral administration was very short. Therefore, the search for new inhibitors of the interaction between progerin and lamin A with optimized pharmacokinetic properties continued until the identification of SLC-D011 or progerinin (Figure 5), a compound able to extend the life span of *Lmna^G609G/G609G^* progeroid mice after oral administration (50 mg/Kg, daily) and to improve histological and physiological hallmarks of progeria in *Lmna^+/G609G^* mice [51,61]. 

#### 2.3.4. Modulators of the Downstream Deleterious Effects Linked to Progerin Accumulation

**Figure 6:**
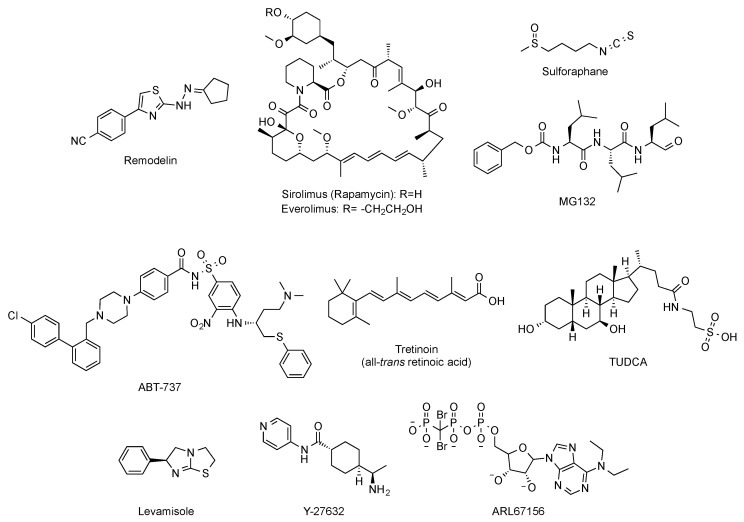
Structure of remodelin, sirolimus (rapamycin), everolimus, sulphoraphane, MG-132, ABT-737, tretinoin (all-*trans* retinoic acid), TUDCA, levamisole, Y-27632, and ARL67156.

Beyond targeted therapies, alternative approaches have addressed the search of small molecules able to ameliorate or reverse the phenotypic harmful effects elicited by the abnormal progerin accumulation in the nuclear lamina. In this regard, and aimed at improving the characteristic nuclear architecture defects of progeroid cells, the development of inhibitors of *N*-acetyltransferase 10 (NAT10) has been explored [62]. In particular, oral administration (100 mg/kg, daily dose) of remodelin (Figure 6), a NAT10 inhibitor that acts in a progerin and FTase independent manner, significantly enhanced the health span of progeroid mice and slowed down the body-weight loss. In addition, remodelin treatment corrected several progeroid hallmarks such as the levels of subcutaneous adipose tissue and the adventitial fibrosis of the aorta. It also reduced the vascular smooth muscle cell loss both in the aorta and the coronary arteries and decreased some of the markers of genome instability in heart and lung of treated progeroid *Lmna^G609G/G609G^* mice [63].

Other strategies have considered the possibility of enhancing the autophagy-mediated progerin clearance. In this way, the use of rapamycin (Figure 6) improved the abnormal nuclear shape, delayed the onset of cellular senescence, and rescued the chromatin phenotype of HGPS fibroblasts [64,65]. This finding led to the consideration of the inhibition of the mTOR (mammalian target of rapamycin) pathway as a pharmacological strategy worth exploring for treating progeria. Aimed at this objective, a variety of studies have established that rapamycin administration (8 mg/kg, intraperitoneal) inhibits mTOR and increases autophagy. The regulation of these signaling pathways translates in vivo into an improvement in cardiac function and in lifespan extension in lamin A/C-deficient mice [66]. These results have been the basis of the phase I/II clinical trial (NCT02579044) of everolimus (Figure 6), a structurally-related analogue of rapamycin, in combination with lonafarnib, which is still ongoing. Other attempts to increase autophagy to promote progerin degradation have explored a variety of molecules such as sulforaphane or MG-132 (Figure 6) [67,68,69].

Global correction of progeroid cellular defects has been also attempted by using molecules with more general mechanisms of action such as antioxidants, reactive oxygen species (ROS) scavengers, and anti-inflammatory or senolytic compounds. For example, the capacity of the ROS scavenger *N*-acetyl cysteine to improve nuclear damage and cell proliferation of HGPS fibroblasts has been reported [70], and analogous effects were observed after administration of the associated protein kinase (ROCK) inhibitor Y-27632 (Figure 6) [71]. Similarly, the use of antioxidants able to improve mitochondrial function has also positive effects in progeroid cells [72]. Additionally, the selective removal of senescent cells with the senolytic compound ABT-737 (Figure 6) has a positive impact on the global senescent signature and in the median survival, which is significantly increased in the heterozygous *Lmna^+/G609G^* progeroid mouse model treated with this compound during their second half of life [73]. 

Phenotypic drug discovery has been also applied to progeria. In this regard, a high-content imaging-based high-throughput screening of hundreds of FDA approved molecules has been carried out, aimed at identifying those compounds that produce an improvement in the structural, epigenetic, DNA damage, and related nuclear defects characteristic of progeroid cells. This study has characterized some retinoids as another class of drugs that can be useful for treating some of the symptoms of the disease [74]. The mechanism of action of these compounds could be mediated by their interaction with the retinoic acid receptor element (RARE) present in the LMNA promoter, thereby repressing lamin A, progerin, and lamin C expression at the mRNA level. In support of this idea, the capacity of all-trans retinoic acid (Figure 6) to decrease the levels of progerin has been reported [75]. Although all these results lay the foundations for deeper studies, in general, up to this moment, the positive effects have been only assessed in cellular models, and the specific molecular targets involved are difficult to determine [76]. The objective of identifying the pathways linked to specific progerin-induced alterations has been successfully addressed in the case of VSMC damage. A recent transcriptomic study has shown that endoplasmic reticulum (ER) stress and the unfolded protein responses play a critical role in the VSMC death characteristic of progeria. Accordingly, administration of tauroursodeoxycholic acid (TUDCA, Figure 6), a chemical chaperon, to two mouse models of HGPS (one with ubiquitous progerin expression and other with VSMC-specific progerin expression) was able to decrease medial VSMC loss and atherosclerosis. In addition, in the VSMC-specific model, TUDCA also increased lifespan [77]. Additional studies have been also focused on the specific correction of some of the most deleterious symptoms of progeria, with a special attention to cardiovascular disease. In this context, reducing vascular calcification by increasing the levels of ATP and pyrophosphate with the administration of the tissue nonspecific alkaline phosphatase (TNAP) inhibitor levamisole and the ectonucleoside triphosphate diphosphohydrolase (eNTPD) inhibitor ARL67156 moderately extended (12%) longevity in the progerin-expressing *Lmna^G609G/G609G^* mouse model [78]. In the same line, dietary magnesium supplementation reduced calcification of vascular smooth muscle cells in vitro and in vivo and improved the longevity of *Lmna^G609G/+^* mice [79]. However, the relevance of this finding to human HGPS remains to be addressed.

## 3. Ongoing Clinical Trials 

All the important advances carried out at the molecular and cellular levels described in the previous sections represent the bases of the ongoing clinical trials, the last step before bench to bedside translation can materialize. In fact, the approval in November 2020 of lonafarnib as a drug for treating progeria was a milestone in the long pathway from the molecular characterization of the disease (in 2003) to the first ever FDA approved treatment for progeria almost twenty years later. Nonetheless, this drug represents a treatment, but not yet a cure for the disease, the far-reaching objective in this quest. Within this final goal in mind, some of the diverse pharmacological approaches already tested in human cells and in the disease mouse models have progressed to the next critical step, i.e., the first time in humans. In progress clinical trials include the phase I/II dose-escalation trial of everolimus in combination with lonafarnib (clinical trial NCT02579044). This study (which is intended to be completed by December 2021) will determine the dose-limiting toxicity (DLT) and the maximum tolerated dose (MTD) of everolimus when administered in combination with lonafarnib, which will be administered to all participants at the doses previously established in the pediatric population. Up to this moment, the majority of the clinical trials have been focused on the administration of lonafarnib, either by itself or in combination with zoledronic acid and pravastatin or with everolimus. Without underestimating the importance of these trials, which have clearly boosted the approval of the first drug for progeria, they are based on the repurposing of molecules already developed for different indications. In this scenario, particularly remarkable is the addition of progerinin (Figure 5) as a first-in-class new candidate specifically developed for progeria. This molecule has started the first-in-human study with a phase I clinical trial (NCT04512963) aimed at determining the safety, tolerability, and pharmacokinetics of progerinin, and it is estimated to be finished by July 2021. 

## 4. Future Perspectives

Less than 10% of rare diseases have an FDA-approved treatment. The usual explanation is that the scarcity of patients discourages pharmaceutical laboratories of undertaking these though challenges based on the perspective of doubtful revenues. In the last decade, some regulatory incentives have been set aimed at expediting the approval process so that working on the development of new therapies for rare diseases can be seen as profitable in the long term. In the case of progeria, the milestone of having a specific approved treatment has just been achieved. Nonetheless, lonafarnib improves some features of the disease, but it does not cure it, which is the current goal of the translational research in this field. Towards this end, a promising landscape lies ahead. With respect to the small molecule field, new first-in-class candidates have been recently described, including ICMT inhibitors and inhibitors of the interaction between progerin and lamin A. The fact that one of them, progerinin, has started phase I preclinical studies is indicative of its promising potential. With respect to biologicals, antisense oligonucleotides have shown encouraging results in progeria, and the efficacy of this approach has been materialized for other diseases. Hence, it is conceivable that optimized candidates with this mechanism of action will be developed in the near future. Going a step forward, the direct correction of the mutation at the gene level by base editing has yielded impressive results in the progeria mouse model. It is true that the use of gene editing technologies in humans has still a long way to go before it can be generally applied; however, it opens an impressive number of possibilities for the treatment of progeria and many other devastating genetic diseases. Finally, the close relationship between progeria and physiological senescence and particularly with respect to the aging-associated cardiovascular complications, which are coincident with the phenotype characteristic of progeria patients, highlights the far-reaching implications of the research carried out in this field. All in sum, and although an extensive workload still lies ahead, it is indubitable that the next years to come will bring exciting new treatment strategies which, either by themselves or in combination with others, will finally provide a definitive cure for this rare disease. 

## Figures and Tables

**Table 1:** Main animal models of progeria.

Model	Main Phenotypic Features	Main Limitations	Ref.
*Zmpste24^−/−^* mice	Bone fragility, reduced weight and growth, defective prelamin A processing, early death, muscular weakness, age-dependent cardiac electrical defects	No severe vascular alterations	[19,20,21]
Heterozygous *LMNA^p.G608G/+^* mice	Ubiquitous progerin accumulation, vascular abnormalities	Lack rest of features of progeria phenotype	[22]
*Lmna^G609G/G609G^* mice	Ubiquitous progerin accumulation, shortened lifespan, reduced weight, main metabolic, bone, and cardiovascular alterations	Mice do not develop atherosclerosis	[23]
*Apoe^−/−^ Lmna^G609G/G609G^* mice	Same phenotype as *Lmna^G609G/G609G^* but including the development of atherosclerosis	-	[25]
*Apoe^−/−^Lmna^LCS/LCS^ SM22αCre* mice	Progerin expression restricted to VSMCs. Mice recapitulate vascular features of progeria	Lack of overt growth defects and other disease symptoms compared to the phenotype observed in *Lmna^G609G/G609G^* mice	[25]
*Apoe^−/−^Lmna^LCS/LCS^ LysMCre* mice	Progerin expression restricted to macrophages	Lack of overt growth defects and other disease symptoms compared to the phenotype observed in *Lmna^G609G/G609G^* mice and in *Apoe^−/−^Lmna^LCS/LCS^ SM22αCre*	[25]
Prog-Tg mice	Progerin expression restricted to endothelium. Reduced growth, weight, and lifespan. Mice recapitulate many cardiovascular alterations such as profibrotic response and cardiac functional impairment	Lack of VSMC loss	[26]
Knockin heterozygous *LMNA c.1824C > T* Yucatan minipig	Expression of progerin and normal lamin A/C, growth retardation, lipodystrophy, skin and bone alterations, cardiovascular alterations, cardiovascular disease, and mortality around puberty	Difficulty to establish an HGPS minipig colony through conventional breeding	[27]
